# Neural development goes retro: Gags as essential modulators of synapse formation

**DOI:** 10.1371/journal.pbio.3003032

**Published:** 2025-02-19

**Authors:** Yung-Heng Chang, Josh Dubnau

**Affiliations:** 1 Department of Anesthesiology, Stony Brook School of Medicine, Stony Brook, New York, United States of America; 2 Department of Neurobiology and Behavior, Stony Brook University, Stony Brook, New York, United States of America

## Abstract

Neurodevelopment requires dynamic control of synapse number. This Primer highlights a new study in PLOS Biology which reveals that the gag protein of Copia, an active retrotransposon, forms virus-like capsids that transfer its own RNA across the Drosophila neuromuscular junction, to regulate synapse formation.

## Teaser

Neurodevelopment requires dynamic control of synapse number. A new study in *PLOS Biology* reveals that the gag protein of Copia, an active retrotransposon, forms virus-like capsids that transfer its own RNA across the *Drosophila* neuromuscular junction. Here, Copia acts antagonistically with Arc, another retrotransposon gag protein, to regulate synapse formation.

The fly larval neuromuscular junction (NMJ) is a powerful model to investigate conserved underpinnings of synapse formation and synaptic transmission, as well as mechanisms of functional and structural plasticity that maintain homeostasis as the size of the NMJ increases during development. In this issue of *PLOS Biology*, M’Angale and colleagues describe a surprising and novel role for retrotransposable elements (RTEs) in synaptic development and plasticity at the larval NMJ [1]. Their findings identify a mechanistic push and pull in which the Copia RTE and the RTE-derived Arc protein act antagonistically to balance the rate of synaptic bouton formation.

RTEs are a subtype of transposable element that replicate through an RNA intermediate. RTEs encode a reverse transcriptase, which synthesizes cDNA from the RTE encoded RNA, as well as protein machinery to re-insert the cDNA copies at de novo chromosome locations. The long-terminal repeat (LTR) subtype of RTEs are evolutionary ancestors of retroviruses [2,3] and share many of the features of retroviral replication, including the fact that they encode a gag protein that assembles a viral-like capsid, bound to its own RNA.

Sequences derived from RTEs make up a vast fraction of the genomes of most plants and animals. Because expression of RTEs has the potential to cause mutations, genomic instability, and activation of inflammatory signaling, organisms have invested heavily in mechanisms to suppress their expression. There is accumulating evidence that when these silencing systems fail, RTEs contribute to diseases such as cancer and neurodegeneration, as well as to the normal aging process [4,5]. Most genomic copies of RTEs have degenerated over time, and are no longer capable of retrotransposition, but many of the RTE proteins and regulatory regions have been exapted to play critical cellular roles [6]. An example of this is the Activity Regulated Cytoskeleton associated protein (Arc), a well-known immediate early gene that plays fundamental roles in synaptic plasticity [7]. It was previously demonstrated that Arc genes of *Drosophila* and tetrapods are derived from exapted gag genes from an ancient and no longer functional Ty3/gypsy family of LTR-RTEs. Importantly, this appears to be an example of convergent evolution: the fly and tetrapod Arc genes were independently exapted from ancient RTE encoded gags. This suggests that in both cases, evolutionarily ancient properties of gag were independently coopted in distant animal lineages [8,9].

Formation of RNA-bound capsids is a fundamental property of RTE encoded gag, which in the case of Arc appears to provide a means for intercellular (across the synapse) transfer of RNA. Indeed, both the fly and mammalian Arc genes retain structural and functional characteristics of RTE and retroviral encoded gag, including the ability to form virus-like capsids, bound to the Arc encoded mRNA. In both mouse and fly, Arc-bound mRNA is released in extracellular vesicles (EVs) which cross the synapse. After transfer across the synapse, the Arc mRNA undergoes activity-regulated translation [8,9]. In flies, Arc has also been shown to promote NMJ structural plasticity, which converges with prior findings on Arc in mammalian synaptic plasticity [9]. The discovery that PNMA2, another Ty3 gag derived gene, has also been coopted for intercellular communication [10], and that membrane targeting of the zebrafish Bik-2 gag is required for neural crest cell migration [11], adds credence to the idea RTE-encoded gags have repeatedly been adapted for intercellular communication. In this issue of *PLOS Biology*, M’Angale and colleagues report the surprising finding that Copia^gag^, one of the proteins encoded by the fully active Copia LTR-RTE, plays an essential role in NMJ structural plasticity.

**Fig 1:**
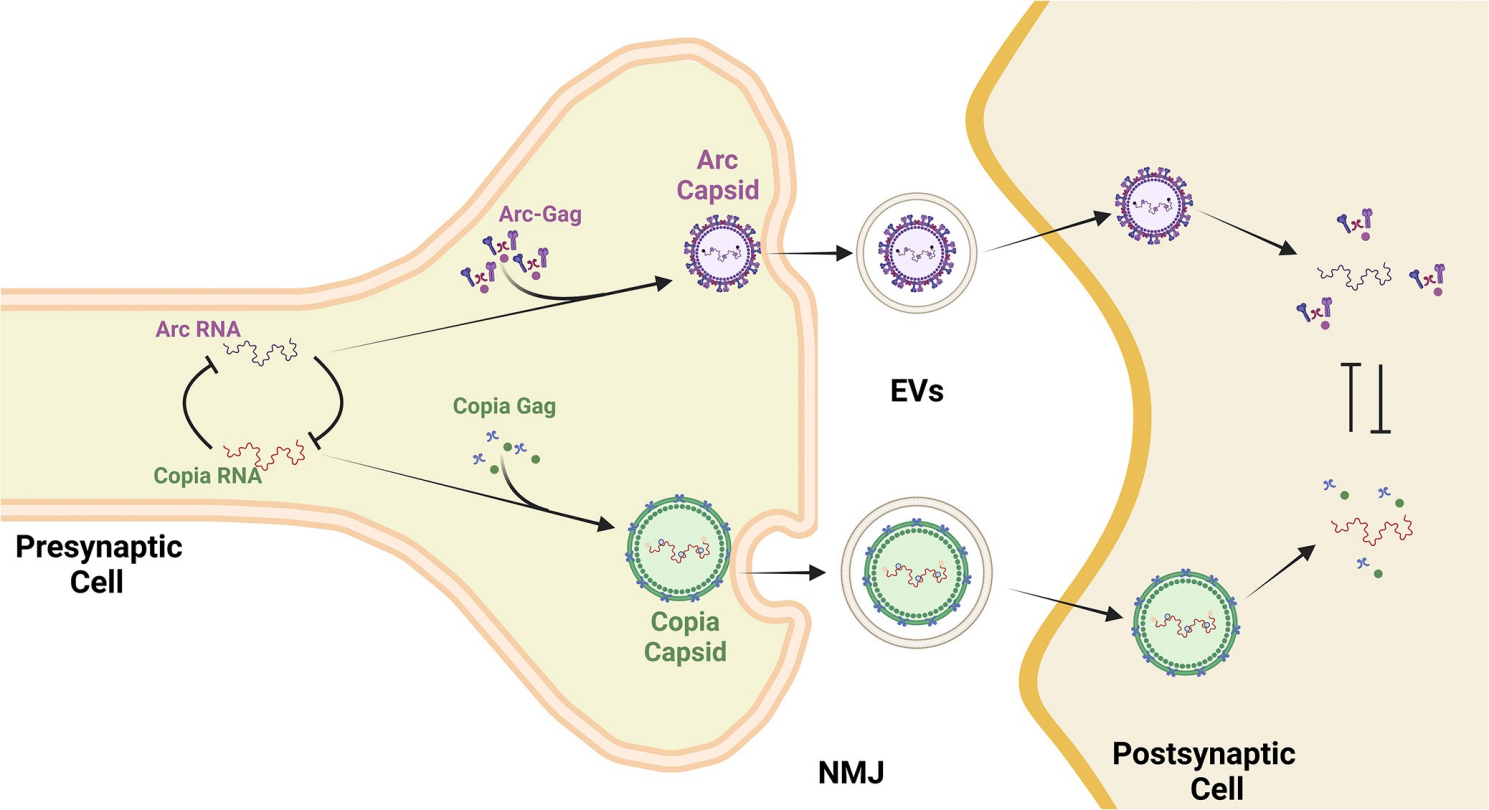
Copia and Arc interactions at the NMJ. In the presynaptic neurons, Copia and Arc RNAs antagonize each other’s expression at the transcriptional level. Presynaptic Copia and Arc RNAs are encapsulated by their own RNA-encoded Gag proteins to form virus-like capsids, which are subsequently released by the presynaptic neurons into the EVs to cross the NMJ. The capsids of Copia and Arc in EVs are then engulfed by the postsynaptic cells, where the virus-like capsids are disassembled to release the embedded RNAs and Gag proteins. The balance of released Copia and Arc RNA and Gag proteins impacts the rate of synapse formation.

Copia^gag^ is essential for Copia replication, and the authors found that purified Copia^gag^ can self-assemble to form virus-like capsids. Using cryo-electron microscopy (cryo-EM), they demonstrate that the nucleocapsid (NC) region, which interacts with RNA, is located deep within the interior of the Copia capsid. The N- and C-terminal domains of the Gag-CA region form the outer shield of the capsid, showing structural similarity to capsids from retroviruses. The authors demonstrate that presence of RNA is crucial for Copia capsid assembly, consistent with its known functional role in Copia retrotransposition. Like Arc, Copia^gag^ is also expressed abundantly in the larval central nervous system and body wall muscles. At the NMJ, Copia^gag^ is detected in both presynaptic neurons and postsynaptic muscle cells. To investigate the source of Copia^gag^, the authors knock down Copia in either pre- or postsynaptic cells, combined with immunostaining to assess the levels of Copia^gag^ present at NMJ. These experiments reveal that at least some of the postsynaptic Copia^gag^ derives from Copia mRNA that is expressed in presynaptic neurons, indicating transfer across the synapse. The Thomson lab previously reported that Copia RNA is loaded into EVs derived from *Drosophila* S2 cells [8]. They now are able to detect Copia transcripts associated with Copia^gag^ capsids in EVs extracted from larval CNS and body wall muscle as well. Together, the findings are consistent with the interpretation that as with Arc, Copia^gag^ bound Copia RNA may be transferred across the NMJ synapse via EVs, leading to translation of the RNA cargo postsynaptically. Investigation of the functional roles of Arc and Copia^gag^ indicates that they antagonistically modulate the number of synaptic boutons (Fig 1).

The Thomson group previously demonstrated that knockout of the fly Arc1 gene leads to a decrease in bouton number [8]. Here, they found that Copia knockdown results in the opposite effect, an increase in the number of synaptic boutons formed. Copia knockdown is also sufficient to sensitize the NMJ to neural activity so that greater structural plasticity is seen from less stimulation. Copia knockdown is also sufficient to ameliorate the loss of boutons seen in Arc1 mutant animals, indicating that Copia’s effects on plasticity are manifested in the absence of Arc protein. These findings indicate that Arc1 and Copia provide a brake and an accelerator on synapse formation, respectively. And yet unlike the brake and accelerator of a car, the opposing effects of these 2 gag proteins are not entirely independent. Rather, they are mutually antagonistic because Copia knockdown leads to increased levels of Arc1 and knocking out Arc1 causes increased Copia expression. Capsid immunoprecipitation experiments demonstrate that although Copia RNA is only detected within Copia capsids, the Arc RNA can be loaded into either Copia or Arc capsids, suggesting a more complex interplay whose impacts are yet to be elucidated.

The discovery of an essential role in neural development for the gag of Copia raises obvious questions about how the functional requirements for Copia^gag^ at the NMJ are balanced against the genotoxic potential from expressing an active and functional LTR-retrotransposon. As more cases of mutualism between active RTEs and the host emerge, this question will need to be investigated. But the repeated examples where gag proteins from LTR retrotransposons have been adapted for use in intercellular trafficking of RNAs is unexpected for another reason [1,8–10]. Phylogenetic analyses of reverse transcriptase sequences indicate that all retroviruses have descended from mammalian LTR-RTEs [2,3]. The emergence of retroviruses from LTR-RTEs involves a change from a strictly intracellular replication cycle of the RTE, in which targeting of capsid assembly is geared towards nuclear entry, to an obligate intercellular replication cycle of a virus. The emergence of retroviruses from their ancestral LTR-RTE cousins required a change in targeting of gag to assemble a virus particle at the plasma membrane for release. Perhaps the repeated adaptation of RTE gag for intercellular trafficking points to a shared evolutionary path with the emergence of retroviruses. Has the host coopted a viral property of gag? Or have retroviruses coopted a property of host gag genes?
